# The Data are Insufficient to Confidently Root the SARS-CoV-2 Phylogenetic Tree

**DOI:** 10.1093/molbev/msaf118

**Published:** 2025-06-09

**Authors:** Jesse D Bloom

**Affiliations:** Fred Hutchinson Cancer Center, Howard Hughes Medical Institute, Seattle, WA 98103, USA

**Keywords:** SARS-CoV-2, MRCA, COVID-19, rooting phylogenetic tree, lineage A, lineage B, Huanan Seafood Market

## Abstract

Several years ago, I published a paper that described the discrepancy between outgroup and date-based methods for rooting the SARS-CoV-2 phylogenetic tree, and proposed the discrepancy could arise from biases among the available early viral sequences. Here, I explain why the root remains uncertain, including via an interactive narrative at https://nextstrain.org/groups/jbloomlab/narratives/SARS2-rooting/early-SARS2-trees-v1 that enables the reader to examine the underlying data and understand discrepancies that lead different methods to reach different inferences about the root. I also demonstrate clear evidence of bias among the earliest available sequences, and explain why the root of the SARS-CoV-2 tree cannot be conclusively resolved with the current data.

## Introduction

Determining the root of the SARS-CoV-2 phylogenetic tree (i.e. which sequence represents the most-recent common ancestor or MRCA of all known sequences) is important for reconstructing the early outbreak. The technical question of how to root the phylogenetic tree has also become entangled with the debate about whether the Huanan Seafood Market was where the virus first entered the human population, or simply an early superspreading event (Cohen 2020; Chen et al. 2020; Huang et al. 2020; Li et al. 2020; Zhang et al. 2020; Pekar et al. 2021; Courtier-Orgogozo and de Ribera 2022; Pekar et al. 2022; Worobey et al. 2022; Bloom 2023; Liu et al. 2024; Bloom 2024; Crits-Christoph et al. 2024; Weissman 2024).

There are two basic principles widely used to infer the root of viral phylogenetic trees. The first principle, *outgroup-based rooting*, is based on the idea that the root of the tree is generally closer to more distant relatives (the outgroup) than later descendant sequences. The second principle, *date-based rooting*, is based on the idea that the sequences with the earliest collection date are generally more similar to the root than sequences collected later. Neither principle is absolute: outgroup-based rooting can be violated if mutations randomly make a virus more similar to outgroup relatives, and date-based rooting can be violated if the available early sequences are a biased subset of all sequences.

For SARS-CoV-2, these two basic rooting principles are in conflict: the first collected sequences (which are mostly from people who visited or worked at the Huanan Market) are not the sequences that are most similar to SARS-CoV-2’s bat coronavirus relatives. This discrepancy was analyzed in detail by Pipes et al. (2021), who concluded *“[t]hese results suggest that phylogenetic evidence alone is unlikely to identify the origin of the SARS-CoV-2 virus and we caution against strong inferences regarding the early spread of the virus based solely on such evidence.”*

In 2021, I published a paper that analyzed rooting of the tree in light of a set of partial SARS-CoV-2 sequences described as being from “early in the epidemic” had been removed from the NCBI’s Sequence Read Archive (Bloom 2021). I argued that doubts about whether available early sequences were fully representative of the early cases should increase the weight given to outgroup-based roots, and suggested two candidates for the root, one of which had been previously proposed by Kumar et al. (2021). Now Débarre and Hensel (2025) argue that other studies have ruled out these two candidate roots. Here, I explain why the data are still insufficient to confidently root the tree, and discuss how biases in the available early sequences and possible reversion mutations could affect efforts to root the tree.

## Results

### The Root of the SARS-CoV-2 Phylogenetic Tree Remains Unclear

To help explain why the root of the SARS-CoV-2 tree remains unclear, I have made an interactive Nextstrain narrative (https://nextstrain.org/groups/jbloomlab/narratives/SARS2-rooting/early-SARS2-trees-v1) that shows different possible roots with annotations of relevant properties (e.g. number of mutations from bat coronavirus ancestor, collection date, etc). I encourage the reader to explore this narrative, which is the most effective way to examine the data. In the rest of this section, I discuss key points within the constraints of static journal figures.

**Fig. 1.**
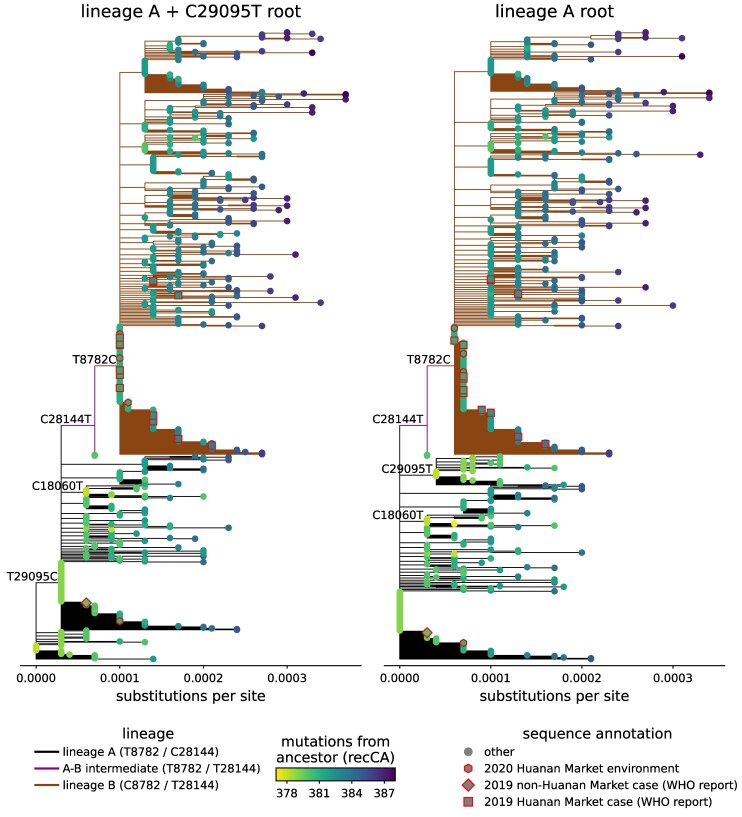
Trees of early SARS-CoV-2 sequences (collection date no later than 2020 February 15) rooted on either lineage A + C29095T (left) or lineage A (right). Sequences (tips) are colored by the number of mutations relative to the inferred bat coronavirus recombinant common ancestor (recCA). Branches are colored according to whether that part of the tree represents lineage A (nucleotides T and C at sites 8,782 and 28,144), lineage B (C8782 and T28144), or a genetic intermediate between lineages A and B (T8782 and T28144). Shapes of tips indicate if the sequence is from a patient with symptom onset prior to 2019 December 31 that did or did not visit the Huanan Market as annotated by the Joint WHO-China study (2021), or from an environmental sample collected from the market on 2020 January 1 by Liu et al. (2024). Key mutations are labeled on internal branches. Note that at the labeled sites, the inferred bat coronavirus ancestor recCA (as well as other relatives like RaTG13 and BANAL-52) have nucleotide identities T8782, T18060, C28144, and T29095. So if the tree is rooted on lineage A + C29095T (left), then the first three trunk mutations (T29095C, C28144T, and T8782C) all make the descendant viruses more different from the ancestor, and C18060T is a reversion towards the ancestor on an internal branch that leads to an appreciable sequence cluster. If the tree is rooted on lineage A (right), then the first two major trunk mutations (C28144T and T8782C) make the descendant viruses more different from the ancestor, and C18060T and C29095T are both reversions on internal branches that lead to appreciable sequence clusters. This tree was built using the sequence set curated by Crits-Christoph et al. (2024). See https://nextstrain.org/groups/jbloomlab/narratives/SARS2-rooting/early-SARS2-trees-v1 for interactive Nextstrain trees that show additional possible roots and similar trees built using the sequence set curated by Lv et al. (2024).

As background, I first explain the “lineage A” and “lineage B” nomenclature that is widely used to refer to early SARS-CoV-2 sequences. SARS-CoV-2 is continuously evolving, and scientists classify related sequences into Pango “lineages.” There are currently >5,000 different named SARS-CoV-2 lineages (see https://github.com/cov-lineages/pango-designation/blob/master/lineage_notes.txt). The first two lineages to be named were called A and B, and differ by just two mutations (at sites 8,782 and 28,144). These lineages are shown in Fig. 1: lineage A has T8782 / C28144, and lineage B has C8782 / T21844. Lineage A is more similar to bat coronavirus relatives (which have T8782 / C28144); however lineage B contains the earliest sequenced human cases from the Huanan Market. This fact was noted in the first scientific paper naming the lineages (Rambaut et al. 2020): *“although viruses from lineage B happen to have been sequenced and published first, it is likely (based on current data) that the most recent common ancestor (MRCA) of the SARS-CoV-2 phylogeny shares the same genome sequence as the early lineage A sequences.”*

The division of early sequences into lineages A and B is an arbitrary classification of the Pango lineage scheme. Lineages A and B differ by only two mutations: branches of two or more mutations are often observed in the phylogenetic tree of a RNA virus with a high mutation rate like SARS-CoV-2, and viral variants that differ by several mutations can arise during human spread in a single community (Tran-Kiem et al. 2025). For a while there was a dispute about whether there were sequences genetically intermediate between lineage A and B (Pekar et al. 2022; Massey et al. 2023), but a study by Lv et al. (2024) confirmed the existence of sequences with a T8782 / T28144 genotype genetically intermediate between lineages A and B.

In fact, neither the defining lineage A nor lineage B sequence are the SARS-CoV-2 sequence most similar to bat coronavirus ancestors/relatives. (Throughout this paper, I will use mutational distances to the inferred bat coronavirus recombinant common ancestor of SARS-CoV-2 termed “recCA” by Pekar et al. (2022), but equivalent results are obtained if mutational distances are referenced to the bat coronavirus relatives RaTG13 or BANAL-52.) Instead, the two appreciable-sized clusters of early (no later than 2020 January) sequences that are closest to SARS-CoV-2’s bat coronavirus ancestor are lineage A + C29095T or lineage A + C18060T (a tree rooted on the first of these genotypes is shown in the left panel of Fig. 1). In Bloom (2021), I proposed that these two sequences represented the most plausible candidates for the root of the tree; Kumar et al. (2021) had previously also proposed the lineage A + C18060T root, and Lv et al. (2024) subsequently also proposed the lineage A + C29095T root. Note that unless there was recombination very early in SARS-CoV-2’s evolution, these two roots are incompatible, so there must have been at least one reversion mutation (either C18060T or C29095T) on a branch leading to an appreciable cluster of sequences early in SARS-CoV-2’s evolution. If the root is instead placed on lineage A, then there must have been at least two reversions (C18060T and C29095T) on branches leading to early clusters of sequences (Fig. 1, right panel). If the root is instead placed on lineage B, then there must have been at least four reversions (C8782T, C18060T, T28144C, and C29095T) on branches leading to early clusters of sequences.

However, while placing the root on lineage A + C29095T or lineage A + C18060T is a good choice from an outgroup-based rooting perspective, neither choice places the root close to most available sequences from the earliest human cases. Specifically, the Joint WHO-China study (2021) states that while there were 174 confirmed cases with symptom onset in 2019 December, just 13 cases with symptom onset prior to 2019 December 31 were sequenced. Of these 13 sequenced cases, 12 visited or worked at the Huanan Market. The sequences from these 12 Huanan-Market-linked cases are lineage B, sometimes with additional derived mutations (Fig. 1). The one sequence from a non-Huanan-Market-linked pre-2019-December-31 case is lineage A, but it lacks C18060T and C29095T and instead contains a derived mutation relative to lineage A (Fig. 1). There are also some sequenced environmental samples collected from the Huanan Market on 2020 January 1 by Liu et al. (2024); most of these are lineage B and the one that is lineage A lacks C18060T and C29095T and contains two derived mutations relative to lineage A (Fig. 1).

**Fig. 2.**
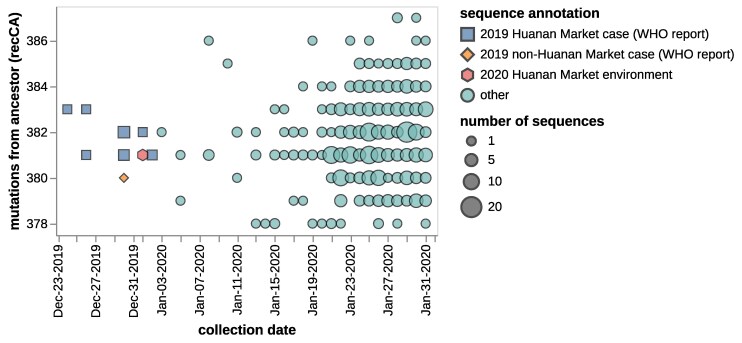
Scatter plot showing the collection date of sequences versus the number of mutations from the inferred bat coronavirus ancestor recCA. Colors and shapes indicate whether sequences are annotated by the Joint WHO-China study (2021) as being from patients with symptom onset prior to 2019 December 31 that had or had not visited or worked at the Huanan Market, or from environmental samples collected from the market on 2020 January 1 by Liu et al. (2024). Shape sizes are scaled according to the number of sequences with that collection and mutational distance. This plot shows sequences collected through 2020 January 31.

Another way to examine the same data is to plot sequence collection date versus the number of mutations from the inferred bat coronavirus ancestor, as shown in Fig. 2. This scatter plot shows how the sequences with the earliest collection dates are not the sequences closest to the bat coronavirus ancestor, and so visually illustrates the discrepancies that led Pipes et al. (2021) to conclude that it was impossible to confidently root the tree.

In their paper, Débarre and Hensel (2025) suggest that the candidate roots of lineage A + C29095T or lineage A + C18060T suggested in Bloom (2021) as well as by Kumar et al. (2021) and Lv et al. (2024) can be dismissed. Débarre and Hensel (2025) support that assertion by citing two earlier papers, Pekar et al. (2022) and Crits-Christoph et al. (2024). However, as summarized in Table 1, a number of papers by various research groups have attempted to the root the SARS-CoV-2 tree. As can be seen by perusing Table 1, these studies reach a range of conclusions about the best candidate for the root, differing even on if it is possible to make a reliable inference. So as is apparent from examining the underlying data (as done above) or reading the diversity of conclusions reached in publications using different well-established phylogenetic methods, there aren’t sufficient data to strongly favor one root over the other possibilities.

**Table 1.** Some studies that have attempted to infer the root of the SARS-CoV-2 phylogenetic tree

Lead and corresponding authors	Citation	Summary conclusions (see citations for full details)
Morel,…, Stamatakis	Morel et al. (2021)	“We cannot draw general, nor confident conclusions about the position of the root using the two mathematically highly distinct approaches that we have deployed here.”
Pipes,…, Huelsenbeck, Nielsen	Pipes et al. (2021)	“These results suggest that phylogenetic evidence alone is unlikely to identify the origin of the SARS-CoV-2 virus and we caution against strong inferences regarding the early spread of the virus based solely on such evidence.”
Kumar,…, Pond, Miura	Kumar et al. (2021)	Suggests root of lineage A + C18060T
Bloom	Bloom (2021)	Suggests root of lineage A + C29095T or lineage A + C18060T
Pekar, Worobey,…, Wertheim	Pekar et al. (2021)	“Our estimates for the timing of the Hubei index case further distance this individual from the outbreak at the Huanan Seafood Wholesale Market.”
Pekar,…, Suchard, Andersen, Worobey, Wertheim	Pekar et al. (2022)	Uses three approaches: **(1)** Date-based (unconstrained) phylodynamic model suggests a root of lineage B; **(2)** Outgroup-based (constrained) model suggests root of lineage A; **(3)** Epidemic model suggests separate introductions of lineage A and lineage B.
Lv,…, Zhang	Lv et al. (2024)	Suggests outgroup-based root of lineage A + C29095T; notes other outgroup-based roots also possible. Confirms existence of sequences genetically intermediate between lineage A and lineage B.
Crits-Christoph,…, Andersen, Worobey, Debarre	Crits-Christoph et al. (2024)	Suggests a root of lineage A; notes that while that root does not correspond to any Huanan Market patient or environmental sequences, those sequences have lineage A as their MRCA.

### Reversions and Biases in Earliest Available Sequences Could Contribute to Discrepancy of Outgroup- and Date-Based Roots

As discussed above, outgroup- and date-based approaches give discordant roots for the tree. It is therefore useful to understand the factors that can confound each approach.

Outgroup-based rooting can be confounded when an evolving virus randomly acquires mutations that make it more similar to its outgroup ancestors/relatives. The probability of this occurring increases with the distance between the root and the sequence(s) being used as an outgroup. The inferred bat coronavirus ancestor recCA differs from the candidate roots of the SARS-CoV-2 tree by ∼380 nucleotide mutations. The SARS-CoV-2 genome is ∼29,000 nucleotide sites in length, and each site can be mutated to 3 other nucleotides. So there are ∼29,000×3=87,000 possible mutations, of which only 380 (or ∼0.4%) make the sequence more similar to the outgroup. However, the chance that a mutation reverts the sequence towards the outgroup are not as low as the naive calculation above suggests. First, only a fraction of the possible mutations are well enough tolerated that we might observe them in a viable sequence, and reversions to the outgroup are more likely to be tolerated than random mutations. Second, some mutation types are more common than others at neutrally evolving sites in the SARS-CoV-2 genome. The most common mutation type is C→T (Bloom et al. 2023), and the additional reversion-to-outgroup mutations that are required when rooting the tree on a sequence other than lineage A + C29095T or lineage A + C18060T are C→T. Furthermore, C→T mutations are more common in some contexts than others (Hensel 2025; Haddox et al. 2025), and C29095T is among the more common C→T mutations. For these reasons, it is possible that C29095T and C18060T reversions could have randomly arisen early in SARS-CoV-2 evolution to create sequence clusters that were more similar to the bat coronavirus ancestor.

**Fig. 3.**
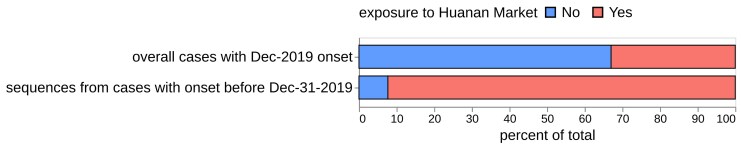
The available earliest sequences are biased towards visitors or workers to the Huanan Market compared to all 2019 December cases. Specifically, according to the Joint WHO-China study (2021), 12 of the 13 sequenced patients with symptom onset before 2019 December 31 visited or worked at the Huanan Market (page 76 of report), but only 33% of all cases from 2019 December are from workers or visitors to the market (page 44 of report).

Date-based rooting can be confounded if the earliest available sequences aren’t actually representative of the earliest cases. In fact, we *know* that the earliest available SARS-CoV-2 sequences are highly biased. Specifically, the Joint WHO-China study (2021) classified most of the 174 lab-confirmed and clinically diagnosed 2019 December SARS-CoV-2 cases by whether the patient visited or worked at the Huanan Market. As shown in Fig. 3, just 33% of these 2019 December cases had recent exposure to the Huanan Market—yet 12 of the 13 of the sequences from pre-2019-December-31 cases (92%) are from workers or visitors to the market. Therefore, the earliest available sequences are not representative of the earliest known cases. Further evidence of this fact can be seen from the fact that the nine *sequenced* patients with earliest symptom onset are market workers or visitors (Joint WHO-China study 2021, page 76), yet multiple independent studies agree that first confirmed cases were not linked to the market (Chen et al. 2020; Huang et al. 2020; Li et al. 2020; Zhang et al. 2020). In Bloom (2021), I argue that the fact that the early sequences may be biased should give increased credence to outgroup-based roots, since they do not depend on the earliest available sequences being representative of the earliest cases.

### Sequences Withdrawn from the Sequence Read Archive

In Bloom (2021), I describe finding that Wuhan University researchers deposited and then withdrew from the NCBI Sequence Read Archive a set of partial SARS-CoV-2 sequences that they described as being from “early in the epidemic.” These sequences were submitted to the Sequence Read Archive in March of 2020, and then withdrawn in June of 2020. When the researchers from Wuhan University requested the sequences be withdrawn, they stated their request was because the data were hard to visit, the data were being updated, and the data had been submitted to another website (FOIA 2022 and sixth figure of Bloom 2021). In fact, the Sequence Read Archive has always been publicly accessible, the sequences have never been updated from the original version, and the data were not submitted to another website until after my preprint posted over a year later in July of 2021. After I posted the preprint of Bloom (2021), the Chinese State Council provided a different reason why the sequences were withdrawn: the journal that published the study accidentally deleted the data availability statement during copy-editing, and this copy-editing error made the researchers think they should delete the actual data (China’s State Council Information Office 2021). In the final version of Bloom (2021), I describe both the explanation given at the time by the researchers and the later incompatible explanation from the Chinese State Council.

Débarre and Hensel (2025) discuss how an Excel table of BioSample-linked collection dates lists 2020 January 30 for all the outpatient samples sequenced in the Wuhan University study, and suggest this makes the sequences not relevant to rooting of the tree. However, all phylogenetic trees and analyses in Bloom (2021) use sequences through the end of January 2020, and in some cases through February. Indeed, this is true of *all* major analyses of the early SARS-CoV-2 tree: as noted above, only 13 sequences are available from patients with symptom onset prior to 2019 December 31, and these patients are biased relative to all cases up to that time (Fig. 3). Therefore, much of the phylogenetic signal in attempts to root the SARS-CoV-2 tree comes from samples collected in January or February of 2020.

The description I use of the samples in Bloom (2021) is a verbatim quote of the Wuhan University researchers, who say in their *medRxiv* preprint (Wang et al. 2020a) that the samples are from *“outpatients with suspected COVID-19 early in the epidemic,”* and in their final published paper (Wang et al. 2020b) that the samples are from *“outpatients with suspected COVID-19 early in the epidemic (January 2020), for whom detailed records and suitable clinical data were unavailable.”* Débarre and Hensel (2025) focus on the dates listed in an Excel spreadsheet in the GitHub repository for my paper that was taken from the supplementary material of Farkas et al. (2020); this spreadsheet lists the collection dates linked to the BioSample for each sequence. As I describe in Bloom (2021), I first identified the withdrawn sequences via that table, and so initially annotated them with the dates in that Excel spreadsheet as that was the only information I had. But after I identified the actual preprint (Wang et al. 2020a) and paper (Wang et al. 2020b) corresponding to the sequences, I updated the annotations in the GitHub repository to match the descriptions in the preprint and paper. Collection date is a required field during BioSample creation. Sometimes researchers provide only approximate BioSample collection dates (see supplementary fig. S1, Supplementary Material online). In fact, this is what was done for at least two of the three sets of samples in the Wuhan University study. Specifically, the respiratory virus sample controls are described by Wang et al. (2020b) as being collected from “November 2019 to January 2020,” but all five have a BioSample collection date of 2020 January 15 (Table 2). The hospitalized patient samples are described by Wang et al. (2020b) as being collected on “February 11 and 12, 2020” but all 16 have a BioSample collection date of 2020 February 12. For the outpatient samples the description is more vague (Table 2), but it remains reasonable to use that description verbatim.

**Table 2.** For each of the three sets of samples in the Wuhan University study, this table lists the number of samples in that set, the collection dates as described in the *medRxiv* preprint (Wang et al. 2020a), the collection dates as described in the final published paper (Wang et al. 2020b), and the collection dates linked to the BioSamples in the Excel table provided by Farkas et al. (2020)

Sample set	Number of samples	Collection date in preprint	Collection date in final paper	Collection date in Excel table
Respiratory virus controls	5	no description	“November 2019 to January 2020”	all listed as “15-Jan-2020”
Hospitalized patients with COVID-19	16	“February 11 and 12, 2020”	“February 11 and 12, 2020”	all listed as “12-Feb-2020”
Outpatients with suspected COVID-19	45	“early in the epidemic”	“early in the epidemic (January 2020)”	all listed as “30-Jan-2020”

After I posted my preprint and the paper was under review, the Chinese State Council stated “According to our understanding, the earliest sampling time of this batch of samples was January 30—some time has passed since the COVID outbreak began. In fact, it is not an early sample” (China’s State Council Information Office 2021). During revision of Bloom (2021), I added to a paragraph noting the description provided by the researchers in their 2020 paper (the samples were from “early in the epidemic”) and the statement by the Chinese State Council that they were “not an early sample.” To be clear, the discrepancy is that the researchers said the samples were “early” whereas the Chinese State Council said that they were “not an early sample.” A 2020 January 30 collection date could be consistent with both descriptions if the researchers and Chinese State Council have different definitions of what constitutes “early.” Note that the BioSample from the CNCB database that (Débarre and Hensel 2025) show in the first figure of their paper was only created in July of 2021, *after* I posted my preprint and submitted my paper.

## Discussion

Phylogenetic inferences are only as good as the data on which they are based. Even the most sophisticated models are maximizing the posterior probability of the model *given the available data* (Bayesian methods), or finding the model that maximizes the likelihood *of the available data* (maximum-likelihood methods).

There are clear reasons to think that the earliest available SARS-CoV-2 sequences are not fully representative of the actual early cases. Multiple studies using different models have reached different inferences about the root of the phylogenetic tree (Bloom 2021; Kumar et al. 2021; Morel et al. 2021; Pekar et al. 2021; Pipes et al. 2021; Pekar et al. 2022; Crits-Christoph et al. 2024; Lv et al. 2024). Here, I have explained limitations of the available sequence data, and created an interactive narrative that enables exploration of these data in a phylogenetic context. Ultimately such exploration is one of the best ways to understand the strengths and weakness of the different proposed roots, since the data limitations that make it impossible to resolve this question solely via phylogenetic models.

## Methods

### Computer Code and Data Availability

See https://github.com/jbloom/early-SARS2-trees for computer code and data used to perform the phylogenetic analyses in this paper and create the interactive Nextstrain (Hadfield et al. 2018) trees and narrative. The GitHub repository contains a full reproducible snakemake (Mölder et al. 2021) pipeline including the input sequence data, except that the sequences used from the GISAID database (Khare et al. 2021) are not included in the GitHub repo due to GISAID data sharing terms, and so must first be downloaded manually using the accessions specified in the relevant CSV files as described in https://github.com/jbloom/early-SARS2-trees/blob/master/data/crits-christoph2024 and https://github.com/jbloom/early-SARS2-trees/blob/master/data/lv2024.

The final interactive trees and the interactive narrative can be rendered via Nextstrain. Specifically, see https://nextstrain.org/groups/jbloomlab/narratives/SARS2-rooting/early-SARS2-trees-v1 for the narrative. The end of the narrative links to trees built with the sequence sets curated by Crits-Christoph et al. (2024) or Lv et al. (2024) with each of the following four candidate roots: lineage A, lineage B, lineage A + C29095T, and lineage A + C18060T.

### Sequences Sets and Phylogenetic Trees

Phylogenetic analyses were done with two different curated sets of early SARS-CoV-2 sequences, those assembled by Crits-Christoph et al. (2024) and those assembled by Lv et al. (2024). The trees linked above and most of the trees in the narrative use all sequences collected no later than 2020 February 15. Interactive trees are also available using all sequences collected no later than 2020 January 31; to access those trees simply replace “Feb-15” with “Jan-31” in the URLs to the interactive trees.

While the sequence subsets of Crits-Christoph et al. (2024) and Lv et al. (2024) are largely overlapping, there are some differences in how they curate, filter, and de-duplicate sequences. In particular, the dataset of Crits-Christoph et al. (2024) removes many of the T8782 / T28144 sequences genetically intermediate between lineages A and B whereas (Lv et al. 2024) do not; whether these sequences should actually all be removed remains a topic of debate (Pekar et al. 2022; Massey et al. 2023; Lv et al. 2024). To be conservative about this unresolved debate, the narrative and paper largely show the trees with the dataset curated by Crits-Christoph et al. (2024) but trees with the Lv et al. (2024) dataset are also made available to allow the reader to assess both.

Due to GISAID data sharing restrictions, neither Crits-Christoph et al. (2024) and Lv et al. (2024) make the actual alignment of sequences they use available, but only provide the accessions. Therefore, the analysis here attempts to rebuild those alignments from the accessions listed in each paper as detailed at https://github.com/jbloom/early-SARS2-trees/blob/master/data/crits-christoph2024 and https://github.com/jbloom/early-SARS2-trees/blob/master/data/lv2024. The precise details of how Crits-Christoph et al. (2024) or Lv et al. (2024) filtered the accessions or attempted to correct sequences for errors purported in later literature but not actually corrected in sequence databases are hard to precisely follow, but https://github.com/jbloom/early-SARS2-trees/blob/master/config.yaml lists under the drop_accessions key specific sequences that were filtered and why. Note that all phylogenetic analyses masked regions 1–222 and 29,700–2,9903 of the genomes, as these terminal regions tend to be poorly sequenced.

To infer the phylogenetic trees, the sequences were aligned using the Nextstrain augur (Hadfield et al. 2018) align subcommand (which uses mafft  Katoh et al. 2002), and all indels relative to the Wuhan-Hu-1 (Genbank NC_045512.2) reference were removed. A tree was then inferred using the augur tree subcommand (which uses iqtree  Minh et al. 2020) with a GTR substitution model. The trees were further refined and annotated using augur subcommands as documented in the Snakefile at https://github.com/jbloom/early-SARS2-trees.

Mutational distances were computed relative to the inferred bat coronavirus recombinant common ancestor recCA of SARS-CoV-2 as reported by Pekar et al. (2022). Distances were also computed relative the bat coronavirus relative of SARS-CoV-2 RaTG13. The figures shown here display distances to recCA, but the interactive figures allow the distances to RaTG13 rather than distances to recCA to be displayed, and the qualitative trends are nearly indistinguishable.

Sequences on the tree were annotated according to whether they were from one of the 13 pre-2019-December-31 symptom onset human cases listed in Joint WHO-China study (2021) (page 76) and if so whether or not that patient had visited the Huanan Market according to that same page of the report, or whether they are from environmental samples collected from the market by Liu et al. (2024) on 2020 January 1.

## Supplementary Material

msaf118_Supplementary_Data

## Data Availability

All code and data are available at https://github.com/jbloom/early-SARS2-trees.
