# Supplementary material for: The Data are Insufficient to Confidently Root the SARS-CoV-2 Phylogenetic Tree
Source: Mol Biol Evol. 2025 Jun 9;42(6):msaf118. doi: 10.1093/molbev/msaf118 (PMC12147021; doi:10.1093/molbev/msaf118)
Supplement: msaf118_Supplementary_Data [file msaf118_supplementary_data.pdf]

Supplementary Material

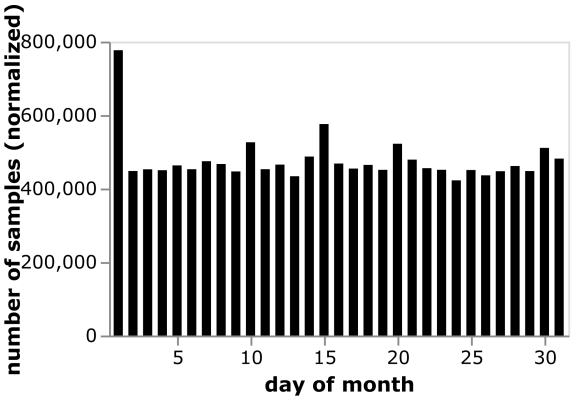

Figure S1 Distribution of days of the month that are reported as the collection dates for BioSamples on the NCBI Sequence Read Archive. Because collection date is a required field, submitters often enter a single approximate date for a large set of samples. The most common date is the first day of the month, with other dates corresponding the 10th, 15th, 20th, and 30th days of the month also over-represented. The number of samples for each day of the month is normalized by dividing by the fraction of months that have that at least that many days. This plot was generated by parsing the XML metadata for all BioSamples in https://ftp.ncbi.nlm.nih.gov/sra/reports/Metadata/NCBI_SRA_Metadata_Full_20250120.tar.gz for the fields `collection date` or `collection_date`.
